# Maintenance and loss of endocytic organelle integrity: mechanisms and implications for antigen cross-presentation

**DOI:** 10.1098/rsob.210194

**Published:** 2021-11-10

**Authors:** Eleanor Childs, Conor M. Henry, Johnathan Canton, Caetano Reis e Sousa

**Affiliations:** ^1^ Immunobiology Laboratory, The Francis Crick Institute, 1 Midland Road, London NW1 1AT, UK; ^2^ Snyder Institute for Chronic Diseases, University of Calgary, Alberta, Canada; ^3^ Comparative Biology and Experimental Medicine, Faculty of Veterinary Medicine, University of Calgary, Alberta, Canada

**Keywords:** endosome, phagosome, membrane damage, membrane repair, cross-presentation, endosomal escape

## Abstract

The membranes of endosomes, phagosomes and macropinosomes can become damaged by the physical properties of internalized cargo, by active pathogenic invasion or by cellular processes, including endocytic maturation. Loss of membrane integrity is often deleterious and is, therefore, prevented by mitigation and repair mechanisms. However, it can occasionally be beneficial and actively induced by cells. Here, we summarize the mechanisms by which cells, in particular phagocytes, try to prevent membrane damage and how, when this fails, they repair or destroy damaged endocytic organelles. We also detail how one type of phagocyte, the dendritic cell, can deliberately trigger localized damage to endocytic organelles to allow for major histocompatibility complex class I presentation of exogenous antigens and initiation of CD8^+^ T-cell responses to viruses and tumours. Our review highlights mechanisms for the regulation of endocytic organelle membrane integrity at the intersection of cell biology and immunology that could be co-opted for improving vaccination and intracellular drug delivery.

## Graphical abstract

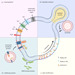

## 1. Introduction

The ability of cells to internalize exogenous material was first observed at the turn of the nineteenth century by Ilya Metchnikoff [1]. He noted that amoeboid cells in starfish larva were capable of ingesting small splinters and termed the process phagocytosis from the Greek words ‘phagos’ and ‘cyte’ meaning ‘to eat’ and ‘cell’, respectively. Nearly half a century later, Lewis [2] recorded a series of time-lapse movies in which phagocytes and transformed fibroblasts could be observed extending wave-like projections over their dorsal surfaces. Occasionally, those structures would recede into the cell to generate large, phase-bright vacuoles filled with the extracellular medium. Lewis termed this process pinocytosis from ‘pinean’ meaning ‘to drink’. Over the years, pinocytosis has been divided into a number of distinct and well-studied processes, including clathrin-mediated endocytosis, caveolae-dependent uptake and macropinocytosis [3,4]. Phagocytosis and pinocytosis allow not only the internalization of exogenous material, but also for the turnover and recycling of plasma membrane constituents. In this review, we focus on events that occur post-internalization, namely those that affect the membrane integrity of endocytic organelles, including endosomes, phagosomes and macropinosomes at different stages of maturation.

After internalization, a series of fusion and fission events occur that are often accompanied by luminal acidification by vacuolar-type ATPases. The concomitant delivery of hydrolases, often with low pH optima, generates an environment that facilitates the degradation of the internalized cargo, which is accentuated by lysosomal fusion. Solutes liberated from the cargo must then be exported out of the vesicle for incorporation into metabolic processes of the cell or for excretion into the extracellular milieu through plasma membrane transporters (reviewed in [4,5]).

The highly dynamic and rapid generation of degradative compartments poses several challenges for the cell, not least of which is to maintain the membrane integrity of these endocytic organelles. The latter must be protected from the harsh biochemistry of the acidic lumen, which can contain membrane-damaging reactive oxygen species (ROS) and degradative enzymes, including lipases and proteases [6–8]. Endocytic organelle membranes must also resist mechanical stresses imposed by the cargo: internalized protein aggregates [9], crystals [10], viruses [11,12], bacteria [13] and hyphae-extending fungi [14] all present distinct physical challenges. Last, but not least, the rapid accumulation of osmolytes from the breakdown of cargo imposes osmotic stress, which, if left unresolved, can rupture membranes [15].

In this review, we will first discuss the mechanisms by which the membrane integrity of endocytic organelles is maintained. Emphasis will be placed on phagocytes, including dendritic cells and macrophages, given their relatively unique ability to continuously endocytose large quantities of exogenous material. Next, we discuss how some cells, specifically dendritic cells, have evolved mechanisms to deliberately rupture endocytic organelle membranes in some instances to perform highly specialized roles in the induction of immunity. Finally, we outline how current and emerging biotechnological and therapeutic applications seek to compromise endocytic organelle integrity to improve delivery of materials to the cytosol of cells.

## 2. The maintenance of endocytic organelle membrane integrity

### 2.1. Osmotic control of membrane tension

One challenge common to all forms of endocytosis is that the osmolyte content of the lumen is, often dramatically, different from that of the cytosol. As endocytic organelles mature, osmolytes gradually accumulate from the breakdown of macromolecules into their building blocks. This is best exemplified during the phagocytosis of apoptotic cells, also known as efferocytosis, which, in effect, doubles the phagocyte load of nucleic acids, proteins, lipids and carbohydrates [16]. The breakdown of such macromolecules into nucleosides, amino acids and sugars generate steep gradients, which, if left unchecked, would result in the movement of water into the lumen, generating outwardly directed tension on the membrane [5]. Biological membranes have a limited capacity for stretch, predicted to be around 3% before they rupture [14,17,18]. To counter osmotically induced tension, an array of transmembrane solute carriers is delivered to endocytic organelle membranes to transport osmolytes into the cytosol (reviewed in [4]). Osmotically induced tension is further reduced by the incorporation of transporters that move monovalent and divalent cations across membranes [5]. The importance of solute carriers can be appreciated from the various lysosomal storage disorders that result from their loss of function [15,19].

**Figure 1.**
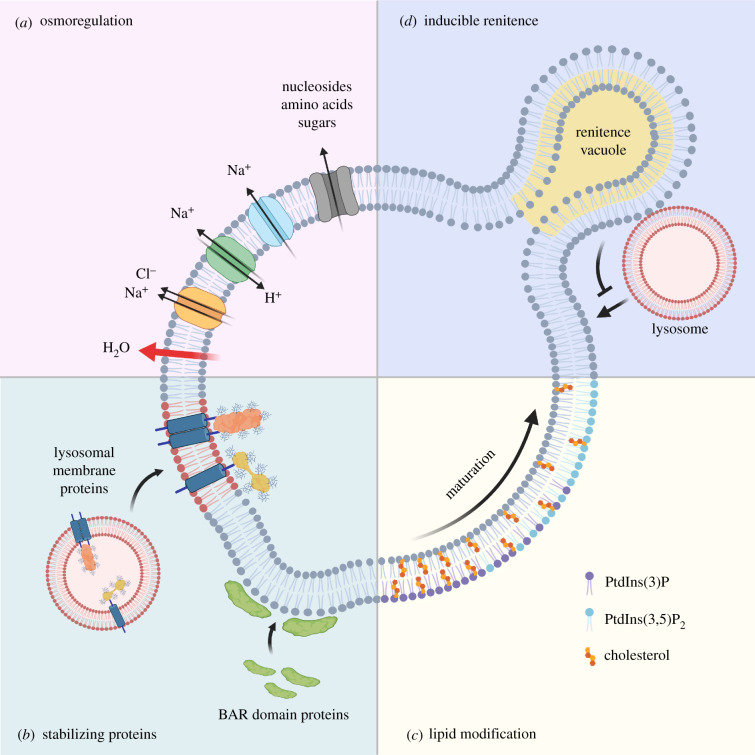
Maintenance of endocytic organelle integrity. (*a*) Following internalization of cargo, osmotically induced tension is regulated by solute carriers and transporters that move monovalent and divalent cations across endocytic organelle membranes. This results in the efflux of water and a subsequent reduction in membrane tension. (*b*) The reduction in volume promotes recruitment of curvature-sensitive BAR domain-containing proteins that stabilize curved membranes, while fusion with lysosomes brings in highly glycosylated transmembrane proteins that help shield the membrane from the luminal environment. (*c*) Modification of lipids regulates organelle trafficking but also facilitates osmoregulation as in the case of phosphatidylinositol 3,5-bisphosphate [PtdIns(3,5)P_2_], which promotes efflux of monovalent ions through lipid-gated channels. (*d*) In macrophages, renitence vacuoles (RVs) are associated with phagosomes and are thought to safeguard against direct fusion between lysosomes and damaged phagosomes. Created with Biorender.com.

The movement of osmolytes across the membrane not only maintains osmotic neutrality but also lowers volume, at least in the case of macropinosomes [20,21] (figure 1). As surface area does not change, the loss of volume results in crenation or furrowing of the limiting membrane, which, in turn, facilitates the maintenance of endocytic organelle integrity by (i) further reducing tension such that any mechanical stress from cargo can be tolerated if necessary through membrane unfurrowing [14,22], and (ii) facilitating the recruitment of membrane-stabilizing proteins via their curvature-sensing domains (discussed below).

### 2.2. Membrane-stabilizing proteins

**Figure 2.**
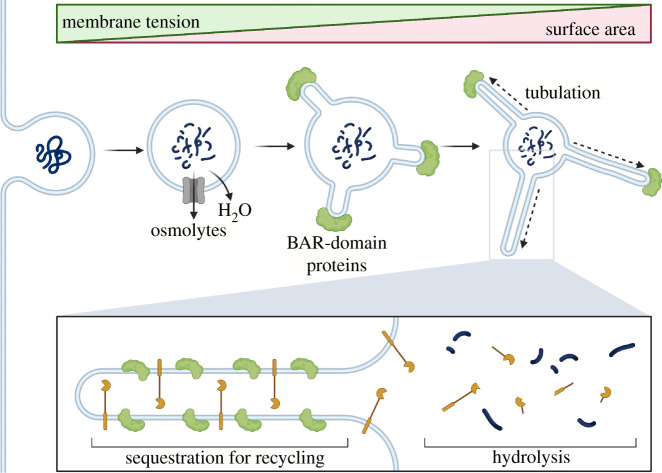
Membrane tubulation induced by BAR domain-containing proteins. Tubulation dependent on BAR superfamily proteins temporarily increases the surface area of limiting membranes to further facilitate osmolyte efflux. It also spatially segregates cargo for recycling away from hydrolytic enzymes. For example, receptors internalized on a macropinosome membrane can be efficiently recycled to the cell surface by tubulation. Created with Biorender.com.

As endocytic organelles lose volume, the reduction in membrane tension results in the recruitment of curvature and tension-sensitive proteins such as those belonging to the Bin–Amphiphysin–Rvs (BAR) domain-containing protein family [5,21,23,24] (figure 1). Although this represents a relatively new area of investigation, BAR domain-containing protein already have well-documented roles in the scaffolding and stabilization of curved membranes [25]. In addition to their direct membrane-stabilizing effect, BAR domain-containing proteins may further buffer against tension-induced membrane stress in a manner akin to caveolae at the plasma membrane [26]. The recruitment of BAR domain-containing proteins also drives membrane tubulation and fission events [27]. These temporarily increase the surface to volume ratio of the endocytic organelle, which likely facilitates the rapid export of osmolytes that are small enough to diffuse into the tubules and access membrane solute carriers [5,28]. Tubulation also helps to spatially segregate cargo destined for recycling (including receptors and membrane) away from hydrolytic enzymes [21,29] (figure 2).

Maturing endocytic organelles can also acquire transmembrane proteins that protect the membrane from the harsh luminal environment [30,31] (figure 1). The best studied are the lysosome-associated membrane proteins LAMP1 and LAMP2 and the lysosome integral membrane protein LIMP2. These proteins are abundant in lysosomes and are delivered to maturing endosomes, phagosomes and macropinosomes through vesicle fusion. As they are heavily glycosylated, they form a thick (approx. 8 nm) luminal glycocalyx believed to physically exclude acid hydrolases [31,32]. Loss of LAMP1 and LAMP2 does not have marked effects on lysosomal membrane integrity [33,34], possibly because of redundancy, but loss of LIMP2 results in severely damaged lysosomes [31].

### 2.3. Lipid bilayer modifications

Just as the protein content of their membranes is modified as endocytic organelles mature, so too is the lipid component (reviewed in [4,35]). Lipid kinases, lipases and efflux proteins can all modify membrane lipids [32,36–38] (figure 1). Lipid modification facilitates the recruitment of proteins involved in endocytic organelle trafficking and maturation but appears to play an additional role in osmoregulation and membrane biomechanics. On phagosomes and macropinosomes, for example, the generation of phosphatidylinositol 3,5-bisphosphate [PtdIns(3,5)P_2_] by the cytosolic phosphoinositide kinase PIKfyve opens lipid-gated channels to facilitate the efflux of monovalent ions and, therefore, water [20,21,38]. This decreases membrane tension, facilitates membrane furrowing and ultimately helps the recruitment of the BAR domain-containing proteins discussed above. PtdIns(3,5)P_2_, incidentally, is also required for the fusion of endocytic organelles with lysosomes and, therefore, for the incorporation of LAMP and LIMP proteins into these organelles [39,40].

Other lipids can have more direct effects on the biomechanics of the endocytic organelle membrane. Cholesterol, a key component of the plasma membrane, is incorporated into the membranes of early endocytic organelles but its levels decrease during maturation [41–43] (figure 1). Cholesterol in the plasma membrane decreases membrane tension and enhances resistance to rupture [44]. Similarly, endocytic organelle membranes enriched in cholesterol are more resistant to damage [45], while lysosomal membranes, which have low cholesterol content, possess the highest amounts of membrane-stabilizing LAMP and LIMP proteins [46]. In addition, cholesterol content is associated with membrane budding events that contribute to volume control of endocytic organelles [47,48]. Nevertheless, exactly how cholesterol contributes to membrane integrity is unclear and, in some cases, cholesterol accumulation in phagolysosomes may actually provoke membrane rupture through formation of membrane-damaging crystals [49,50].

### 2.4. Renitence vacuoles

Professional phagocytes employ unique and inducible mechanisms to guard against phagosomal damage. Macrophages form so-called renitence vacuoles (RVs) during phagocytosis as they take up particulate targets [51,52]. RVs form in a process much like macropinocytosis but differ in that they do not shrink in size as they mature. Rather, they remain associated with phagosomes and buffer the fusion with lysosomes via a mechanism that remains unclear [52] (figure 1). RV formation is enhanced upon stimulation of phagocytes with microbial products such as lipopolysaccharide and is believed to safeguard against lysosomal fusion with phagosomes damaged by pathogens [52].

## 3. Repair and removal of damaged endocytic organelles

**Figure 3.**
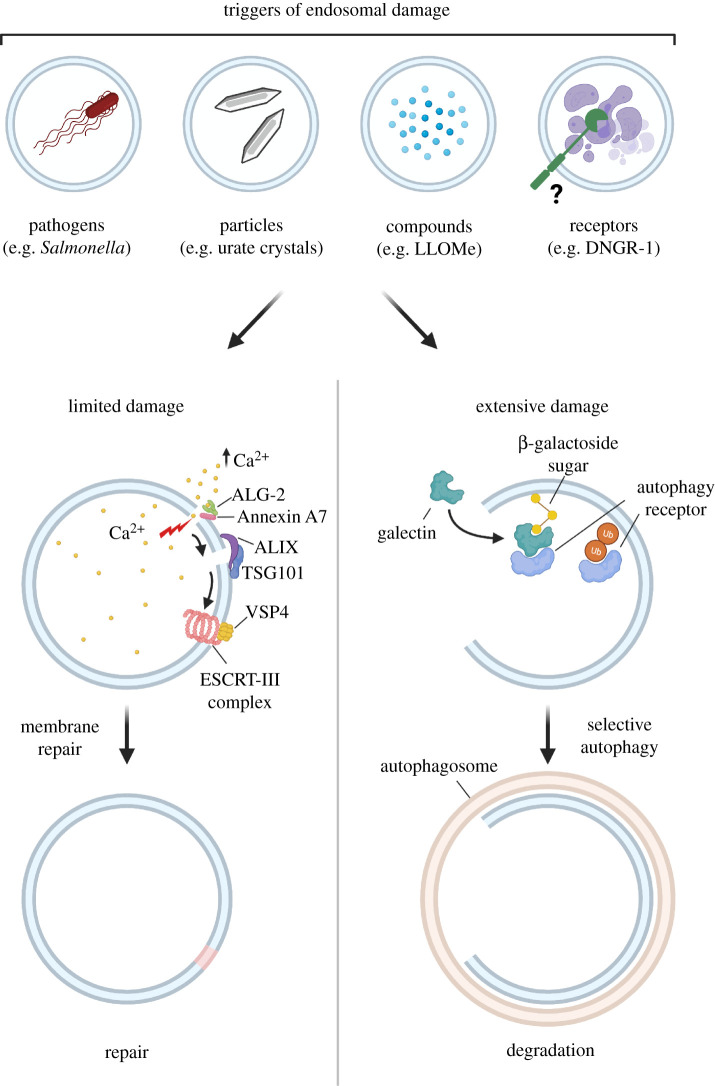
Repair and removal of damaged endocytic organelles. Internalization of some agents can inflict damage to endocytic organelle membranes, leading to rapid Ca^2+^ efflux. Elevated cytosolic Ca^2+^ is likely sensed by ALG-2/PDCD6, which recruits components of the ESCRT machinery such as ALIX and TSG101, culminating in recruitment of ESCRT-III (e.g. CHMP4B, VPS4) to seal the damaged membrane. In cases of more extensive membrane damage, galectins (-3/-8) bind exposed β-galactoside residues on luminal proteins and provide a recruitment signal for cargo receptors such as NDP52. Cargo receptors can also recognize ubiquitin chains on the damaged membrane. These receptors trigger the formation of LC3-containing autophagic membranes, leading to removal of the damaged organelle via selective autophagy. Created with Biorender.com.

Despite the barriers discussed above, damage to the membrane of endocytic organelles does occur, often induced by foreign agents such as pathogen-derived toxins or engulfed particulates [53,54] (figure 3). As such, eukaryotic cells have evolved mechanisms for rapid repair of membrane damage using a highly ordered sequence of interconnected events that rapidly stabilize the injury, remove damaged lipids and replace the damaged membrane [55,56]. This was explored initially in the context of damage to the plasma membrane [55–57] but has since been extended to the repair of endocytic organelle membranes [53,54,58,59] (figure 3).

### 3.1. Signals for endocytic organelle repair

A universal signal for membrane repair is the localized flux of calcium ions (Ca^2+^) at sites of membrane damage [56]. Eukaryotic cells maintain a steep concentration gradient of Ca^2+^ across the plasma membrane and sudden rupture results in localized influx of Ca^2+^ ions. This activates a diverse range of proteins such as calpains, transglutaminases, synaptotagmins and components of the SNARE fusion complexes that can function in membrane repair [57,60]. Cells deficient for calpain-I have impaired plasma membrane repair responses [61]. Calpain-I proteolytically degrades exposed cortical cytoskeletal substrates such as talin and vimentin at the site of injury, denuding the damage site and thereby facilitating fusion of exocytic vesicles that repair the membrane [61]. Calcium-activated transglutaminases mediate protein cross-linking and serve to limit diffusion across the injury site [57,60]. Calcium-regulated exocytosis of endocytic organelles, such as lysosomes, is also thought to be essential for plasma membrane repair. Such calcium-dependent exocytosis of lysosomes is regulated by the lysosomal membrane protein synaptotagmin-7 through its interactions with the v-SNARE VAMP7 on lysosomes and t-SNARE syntaxin-4 on the plasma membrane [57,60].

### 3.2. Re-sealing broken membranes with ESCRT

Lesions of less than approximately 100 nm are typically repaired by components of the endocytic sorting complexes required for transport (ESCRT) machinery (figure 3). The ESCRT complex is responsible for many key membrane remodelling processes including intraluminal vesicle formation, cytokinetic abscission, exosome release and viral budding [62]. Following damage with membranolytic agents, escape of Ca^2+^ from endocytic organelles into the cytosol [54] causes recruitment of both ESCRT-I proteins TSG101 and ALIX, which in turn recruit the ESCRT-III complex to promote rapid re-sealing of the lesions [53]. ESCRT repair of damaged endocytic organelle membranes is thought to occur through exosomal shedding or ‘budding off’ of the damaged lipids [53,54]. The depletion of ESCRT components prevent this shedding and results in death of the cell [53,58]. Intriguingly, an ESCRT-independent repair pathway involving trans-bilayer motion of sphingomyelin on damaged lysosomes has also, recently, been reported [63].

### 3.3. Removal of damaged endocytic organelles by selective autophagy

Larger fissures cannot be repaired by ESCRT-dependent mechanisms and tend to trigger the removal of damaged endocytic organelles via selective macroautophagy (hereafter referred to as autophagy). In this process, the damaged endocytic organelle is specifically recognized, sequestered within an autophagosome and ultimately recycled via lysosomal degradation (figure 3). Various forms of selective autophagy exist, of which lysophagy (the selective degradation of lysosomes) [64] and xenophagy (the selective degradation of intracellular pathogens, literally ‘foreign-eating’) [65] are particularly pertinent for this review.

Damaged phagosomes and endosomes appear to undergo selective removal via mechanisms similar to lysophagy. The process of selective autophagy of endocytic organelles is initiated by galectins, a family of lectins predominantly localized within the cytosol. Galectins bind to β-galactoside-containing glycoconjugates that would normally be restricted to the extracellular aspect of the plasma membrane or the lumen of endocytic organelles but can become exposed to the cytosol upon damage [66]. The role of galectins in the detection of damaged endocytic organelles was first discovered as a response to ruptured vacuoles during bacterial invasion [67,68] but they are now known to be involved in the cellular response to diverse triggers of endomembrane damage, including viruses, bacterial toxins, crystalline particles, neurotoxic protein aggregates and transfection reagents [69–72]. The recruitment of galectins to damaged endocytic organelles occurs within minutes and detection of this phenomenon by immunofluorescent staining with anti-galectin antibodies or by using fluorescent galectin fusion proteins is a common method to identify membrane disruption [73].

Of the 15 mammalian galectins, galectin-3 and galectin-8 are best-established as initiators of damaged endocytic organelle autophagy. Galectin-1 has also been shown to participate in lysophagy and xenophagy of some but not all invasive bacterial species [70,74], although it and galectin-4 are only weakly recruited to damaged endocytic organelles [75]. This is likely due to compartment-specific differences in membrane glycan composition as the fine specificity of galectins varies despite the fact that they all have affinity for β-galactosides [76]. This specificity may also drive the recruitment of different galectins to distinct microdomains on damaged endocytic organelles [77].

Galectins initiate selective autophagy through recruitment of a number of cytosolic autophagy receptors, including p62, nuclear dot protein 52 (NDP52), optineurin and TAX1BP1 [70,78–80], which direct galectin-decorated damaged endocytic organelles to the autophagy machinery. Autophagy receptors also recognize ubiquitin that has been added by E3 ubiquitin ligases onto proteins (or lipids as recently discovered in the case of *Salmonella* [81]) at sites of endocytic organelle damage. The recruited autophagy receptors tether endomembrane damage sites to ATG8 paralogues, such as LC3, on autophagosomal membranes, sorting the damaged endocytic organelle for degradation (reviewed in [82,83]).

### 3.4. Coordination between repair, removal and biogenesis

Membrane repair by ESCRTs and selective autophagy of injured endocytic organelles have been considered largely distinct. However, although accumulation of the ESCRT component ALIX on damaged lysosomes is initially Ca^2+^-dependent and galectin-3-independent [53,54,84], galectin-3 appears essential for enhancing the recruitment of both ALIX and the ESCRT-III component CHMP4 for restoration of lysosomal function following damage [84]. Furthermore, at later stages of the damage response, interactions between galectin-3 and the ubiquitin ligase TRIM16 promote a switch from membrane repair to autophagic removal of damaged lysosomes [84]. The core autophagy-related protein ATG9A has also, recently, been reported to interact with multiple ESCRT components and cooperate with the ESCRT machinery in plasma membrane sealing after damage [85], although whether a similar phenomenon occurs in the membrane repair of endocytic organelles is unknown. Finally, galectins also complement the loss of damaged lysosomes by stimulating lysosome biogenesis through the activity of the transcription factor TFEB [84,86]. Activation of lysophagy also results in small LAMP2^+^ vesicles accumulating in the cytosol, perhaps enabling recycling of LAMP2 [87]. These observations suggest that damage elicits a coordinated response designed to restore endomembrane homeostasis.

## 4. Phagosomal rupture and cross-presentation

There are instances when endocytic organelle rupture might be beneficial and even actively promoted. Presentation of antigens as peptides bound to major histocompatibility complex (MHC) proteins is a key mechanism by which antigen-presenting cells (APCs) orchestrate antigen-specific T-cell immunity [88,89]. MHC class I (MHC-I) predominantly presents fragments of proteins synthesized by the cell (endogenous antigens), effectively displaying at the plasma membrane a representation of that cell's proteome for perusal by CD8^+^ T cells. However, in order to induce effector CD8^+^ T cells against tumours and some viruses, APCs, such as dendritic cells, additionally need to present exogenous antigens on MHC-I [89,90]. This is known as ‘cross-presentation’ (XP) and its mechanistic basis has been the subject of intense scrutiny since its discovery more than 40 years ago [91]. Two main mechanisms have been proposed. In the first, luminal proteases degrade antigens into peptides that directly bind MHC-I present in phagosomes [92,93] (reviewed in [94]). The second proposes that exogenous antigens undergo phagosome-to-cytosol (P2C) transfer to effectively become ‘endogenous’ antigens. This has been postulated to occur via specific transporters that translocate polypeptides to the cytosol [95,96] or by membrane disruption that permits wholesale disgorgement of phagosomal contents [97,98]. The latter has gained experimental support in recent years [99–101] revealing a link between inducible phagosomal rupture and immunity.

A possible connection between disruption of endocytic organelle membranes and cross-presentation was first proposed as the ‘indigestion’ hypothesis. It suggested that a subset of phagosomes release their content into the cytosol in a stochastic manner, allowing access of exogenous antigens to the endogenous MHC-I processing and presentation pathway [97,98]. While the experiments leading to the ‘indigestion’ hypothesis used inert particles, the notion that vacuolar compartment rupture associates with cross-presentation has also been noted during infection. For example, *Mycobacterium tuberculosis* or *Listeria monocytogenes* containing the pore-forming haemolysin, listeriolysin O, can induce permeabilization of vacuolar membranes to allow bacterial and other exogenous antigens to be presented by MHC-I [102–104]. Thus, bacterial-induced P2C correlates with MHC-I cross-presentation but whether P2C can be actively induced by the phagocyte and extends to non-infection scenarios has only been explored more recently.

**Figure 4.**
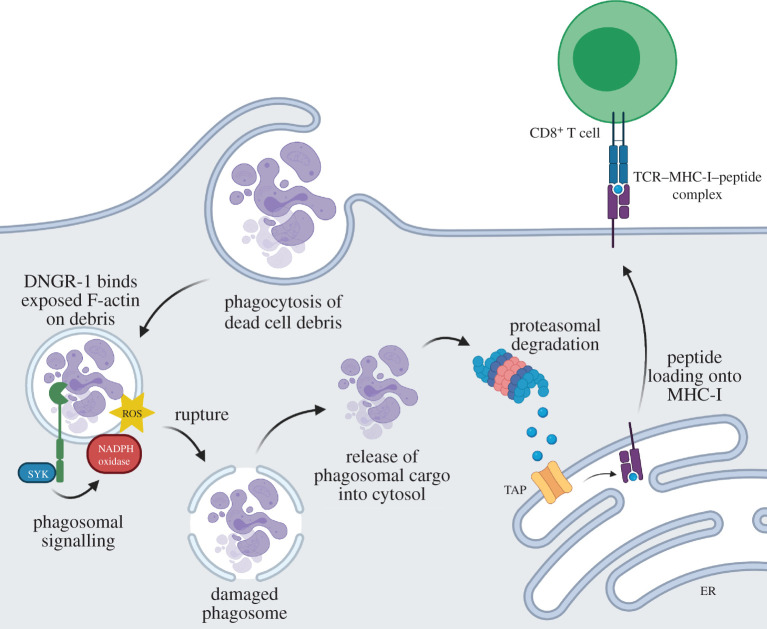
DNGR-1 signals to potentiate ROS-dependent rupture of endocytic organelles and favour P2C and XP of dead cell-associated antigens. In dendritic cells expressing DNGR-1, recognition of F-actin exposed by dead cell cargo within endocytic organelles induces DNGR-1 signalling through SYK, leading to NADPH oxidase activation. ROS produced by the NADPH oxidase damages organelles to increase the probability of rupture and release of cargo into the cytoplasm. There, dead cell-associated antigens are degraded by the proteasome machinery and resulting peptides trafficked into the endoplasmic reticulum (ER) via the TAP transporter for loading onto MHC-I molecules. Peptide–MHC-I complexes are then transported to the membrane and presented to CD8^+^ T cells. Created with Biorender.com.

An important source of antigens for cross-presentation is dead tumour or virally infected cells that can be phagocytosed by dendritic cells [105–107]. Dendritic cell subtypes that take up dead cell debris often express the receptor DNGR-1 [107], which binds F-actin exposed by the cargo [108,109]. We have, recently, found a role for DNGR-1 in promoting phagosomal damage to induce cross-presentation of dead cell-associated antigens [101]. We found that in response to ligation by F-actin exposed in dead cell remnants, DNGR-1 in phagosomes signals via the kinase SYK to induce intense and sustained local activation of the NADPH oxidase protein complex, which, in phagocytes, includes the NOX2 catalytic subunit. NADPH oxidase activation induces ROS and increases the probability that some of those phagosomes will rupture and permit release of cargo (figure 4) [101]. Notably, the DNGR-1-SYK signalling axis can induce phagosomal membrane rupture and cross-presentation in heterologous cells, including non-professional APCs such as HEK293T cells that have NADPH oxidases, suggesting that the pathway is latent in multiple cell types but requires specialized receptors to ‘plug’ into it. Notably, SYK is also the adaptor kinase for a large number of immunoreceptors including Fcγ receptors, integrins, C-type lectin receptors, the B cell receptor and adaptors such as DAP12 and FcRγ [110]. It is interesting to speculate that additional receptors that signal via SYK might in some instances couple to the phagosomal damage pathway described above, perhaps by engaging the SYK effector, VAV. This might explain instances of cross-presentation following phagocytosis of different particle-associated antigens, which has in some cases been shown to involve a Vav–Rac–NOX2-dependent cross-presentation pathway [111,112].

Precisely how NADPH oxidase activation within the phagosome contributes to phagosomal rupture is poorly understood. ROS produced by NADPH oxidase can directly damage endocytic organelle membrane lipids to increase membrane fragility and the probability of rupture [99,100]. Oxidized lipids alter the physical properties of the lipid bilayers disturbing ion transport, increasing membrane permeability, promoting aberrant membrane curvature and decreasing lateral diffusion of lipids [113,114]. Of note, lipid peroxidation of the phagosomal membrane has, recently, been reported by van den Bogaart and colleagues [99,100] to promote antigen release from endosomes and promote cross-presentation.

Synchronized rupture of phagosomes and the release of destructive lysosomal proteases into the cytoplasm is likely to be toxic and cause cell death [115]. As DNGR-1 is confined to the early endocytic pathway, its signalling only increases the rupture probability of early LAMP1^−^ non-degradative phagosomes. This remains a stochastic process and is possibly offset by repair via ESCRT-III such that only a subset of early phagosomes may rupture in any given DNGR-1-expressing dendritic cell at any given time. This may serve to balance the need for cross-presentation of dead cell-associated antigens with preventing overwhelming cell toxicity. Following DNGR-1 engagement, sensors of membrane damage including galectins accumulate on the ruptured phagosomes through binding to exposed glycans present on the intraluminal side of the phagosomal membrane [101]. As discussed above, galectins orchestrate coordinated programmes of repair, removal or replacement of damaged endo(lyso)somal compartments [70,84,86,116]. Whether ruptured phagosomes undergo autophagic removal remains an open question.

## 5. Applications of endocytic organelle disruption in medicine

Just as phagosomal rupture may be useful in cross-presentation, disruption of endocytic organelle membranes provides a framework for the delivery of therapeutics to intracellular targets. Many disease-relevant proteins are cytoplasmic and, as such, overcoming the rate-limiting step of exiting the endocytic organelle to reach them remains a key priority for delivery of drugs that cannot diffuse passively across membranes. A range of strategies to induce escape have been proposed, including mechanical disruption [117], fusion [118,119], pore formation [120], osmotic pressure [121], ROS [122], phospholipase activity [123], molecular transporters [124] and vesicle budding and subsequent collapse [125]. However, translational success has been limited [124,126,127]. Encapsulation within lipid nanoparticles has proven successful in recent years for first-in-kind RNA-based therapies, notably the siRNA therapeutic patsiran (Onpattro) approved by the FDA in 2018 for the treatment of hereditary amyloidogenic transthyretin amyloidosis [128] and the novel class of mRNA-based SARS-CoV-2 vaccines developed for the COVID-19 pandemic [129]. These lipid nanoparticles typically comprise an ionizable cationic lipid thought to interact with negatively charged bilayer lipids to destabilize endocytic organelle membranes, and three helper lipids (cholesterol, a PEGylated lipid and a structural lipid such as distearoylphosphatidylcholine) that improve stability and augment endocytic organelle escape [130]. Mechanistic understanding of the latter process may well enable more successful development of new delivery systems [127]. The finding that DNGR-1 signalling via SYK and NADPH oxidase promotes P2C [101], for example, suggests a pathway that could be exploited to enable delivery of therapeutic macromolecules into the cytosol.

Drug delivery mechanisms will likely need to be optimized depending on the cargo, endocytic route and targeted cell type [124,131]. Overall, activation of membrane disruption activity during early endocytic stages would be preferable in most applications in order to avoid the release of lysosomal proteases that may trigger cell death [115]. Conversely, transformation is frequently characterized by changes in lysosomal membrane volume and composition, rendering cancer cells more vulnerable to lysosomal membrane destabilization [132]. Agents targeting lysosomal membranes may exhibit preferential cytotoxicity in tumour cells and be advantageous as anti-cancer drugs.

## 6. Concluding remarks

Maintenance of endocytic organelle integrity is critical for normal cellular function. Whether in the steady-state, as a consequence of invasion by intracellular pathogens, or because of ingestion of particulate matter, cells that are unable to maintain endocytic organelle integrity typically die. Here, we have summarized multiple strategies used by cells to maintain the integrity of their endocytic compartments. These include regulating membrane tension through osmotic pumping, alterations in lipid composition and recruitment of membrane-stabilizing proteins, while in parallel repairing minor damage or, when damage is extensive, recruiting components of the autophagic machinery to coordinate disposal of broken endocytic organelles. We have placed emphasis on phagocytes and indicated how their response to endocytic organelle damage can also differ depending on the nature of the cell and the cargo. In macrophages, the rapid and coordinated repair or removal of phagocytic or macropinocytic vacuoles containing microbes can be understood from the perspective of limiting pathogen dissemination. In dendritic cells, signalling to increase phagosomal damage may provide a means of coupling recognition of ‘antigenically interesting’ cargo to the cross-presentation pathway. We suggest that regulation of endocytic organelle damage remains an area for future discovery in the fields of cell biology and immunology with applications for drug delivery.

## Data accessibility

This article has no additional data.
